# Characterization of Polycarbonate and Glass-Filled Polycarbonate Using Multi-Relaxation Test—Role of Glass Fiber on Viscous Behavior of Matrix in Fiber Composites

**DOI:** 10.3390/polym17111469

**Published:** 2025-05-26

**Authors:** Jingchao Wang, P.-Y. Ben Jar

**Affiliations:** Department of Mechanical Engineering, University of Alberta, 10-203 Donadeo Innovation Centre for Engineering, 9211-116 Street NW, Edmonton, AB T6G 1H9, Canada; ben.jar@ualberta.ca

**Keywords:** glass fiber, polycarbonate, relaxation, modeling, viscous deformation

## Abstract

The work presented here describes an approach that separates the viscous stress from the quasi-static counterpart for polycarbonate (PC) and its short glass fiber composite (GF-PC), with the aim to characterize the influence of short glass fiber on the viscous behavior of PC as the matrix of GF-PC. A multi-relaxation (MR) test was used for the mechanical testing and a three-branch spring–dashpot model for the data analysis, using a genetic algorithm to establish 100 sets of fitting parameter values that enabled the three-branch model to regenerate the measured stress decay during relaxation. Using the spring modulus $\left(K_{v,s}\right)$ of the short-term branch in the three-branch model, two groups for these fitting parameter values were established as a function of specimen displacement (named stroke) of GF-PC, one of which shows a trend that is similar to the trend of the corresponding fitting parameters for the pure PC, and thus is believed to reflect the influence of glass fiber on the PC matrix of GF-PC. The study concludes that the short glass fiber increases the short-term viscous stress, but its role on the long-term viscous stress is marginal.

## 1. Introduction

Fiber has long been used to increase the strength and stiffness of polymers, especially for the load-bearing applications. Since polymers are known for their viscous properties, their long-term deformation is difficult to predict using the short-term test results. Therefore, extensive studies have been dedicated to understanding the viscous behavior of polymers so that models based on short-term test results could be used to predict long-term behavior. Such studies have also been extended to polymer-based fiber composites. Since fiber composites require reliable long-term mechanical performance, it is important to understand the influence of fiber on the viscous part of the mechanical properties. The work presented here is concerned about the role of short glass fiber on the mechanical properties of glass-fiber-reinforced polycarbonate (GF-PC), focusing on the influence of the glass fiber on the viscous stress response of the polycarbonate (PC) matrix in GF-PC.

PC is an attractive thermoplastic polymer that has been increasingly popular for applications in construction, automotive and electronic industries due to its high stiffness, excellent impact resistance and light weight compared to the conventional materials for these applications. GF-PC is a short-glass-fiber-reinforced PC for further improvement of strength, stiffness and toughness in order to meet the increasingly stringent requirements for these applications.

Numerous studies have been conducted to characterize pure PC and GF-PC using experimental testing and modeling. These studies often characterize pure PC and GF-PC using short-term mechanical tests by applying uniaxial tensile, impact and indentation loading [1,2,3] to evaluate the mechanical performance. It is now widely accepted that the incorporation of glass fiber in PC increases the strength and Young’s modulus [4,5] and improves the creep and relaxation resistance [6,7,8,9]. The short-term creep test has also been used to construct a master curve of creep compliance as a function of time for the evaluation of the influence of fiber content on the creep behavior at different time scales [10]. However, these studies are not able to quantify the role of glass fiber on the viscous stress response to deformation, not only because the traditional tests for this purpose take a long time to complete and thus cannot provide a sufficiently large amount of data in a reasonably short period to provide a statistically reliable evaluation, but also because deformation mechanisms generated in the short-term tests may not include the mechanisms that dominate the deformation in the long-term behavior. However, if an approach is available to separate the viscous deformation mechanisms from the quasi-static counterpart, then it would then be possible to use the short-term tests to evaluate the long-term performance. This concept is examined here using GF-PC and PC as a sample material system to evaluate the feasibility of distinguishing the influence of glass fiber on the viscous part of the stress response to deformation from the quasi-static counterpart.

Models in the literature that separate the viscous stress response to deformation from the quasi-static counterpart are largely categorized into two distinctive groups: the microstructure models [11,12,13] and the spring–dashpot models [14,15,16]. The spring–dashpot models are popular because they can accurately replicate the stress response determined from the mechanical tests to provide a clear visual of the time-dependent deformation behavior, while the microstructure models are used to predict the stress response based on the microstructural change. However, to our knowledge, the microstructure models reported so far in the literature could not mimic the deformation behavior as closely as the spring–dashpot models. One major concern for the spring–dashpot models is that they do not always provide a unique set of values for the fitting parameters. As a result, spring–dashpot models have rarely been used to establish a relationship between microstructural changes and deformation behavior. We believe that such a relationship should exist and could be established using spring–dashpot models if the variation of the fitting parameter values is considered in the establishment of the relationship. As a result, one of the objectives of the current study is to establish the range of fitting parameter values that could regenerate the test results. Based on the trend of changes of these fitting parameter values during the deformation process, the other objective of the study is to examine the possibility of using these fitting parameters to establish the relationship between the fitting parameters and the microstructural change in the deformation process.

The search for the value range of the fitting parameters was through a genetic algorithm that is based on a natural selection process for biological evolution [17]. Previous works have shown that this approach has a high efficiency in compiling a comprehensive set of the fitting parameter values for a variety of deformation behaviors, including visco-elastic and visco-hyper-elastic deformation of soft tissues [18,19], visco-plastic deformation of high-density polyethylene (HDPE) [20] and visco-hyper-elastic deformation of rubber-like materials [21]. Furthermore, the modified versions of the genetic algorithm, such as the real-coded genetic algorithm (RCGA) [22] and the hybrid optimal algorithm that merges with the Broydon–Fletcher–Goldfarb–Shanno search [23,24], have shown the significant improvement in the quality and efficiency for determining the fitting parameter values for elastic–visco-plastic and visco-elastic models, respectively. However, most of the studies in the literature only presented one set of the values for the fitting parameters. It was not clear whether this one set of fitting parameter values could represent the general trend of variation for these fitting parameters. Note that a previous work from our group [25] has reported ten sets of fitting parameter values for a three-branch model, all of which could closely mimic the stress response of HDPE to deformation. However, the ten sets of values might not be sufficient to represent all possible values for these fitting parameters.

The work presented here is to determine one hundred sets of fitting parameter values, all of which could enable the three-branch model to regenerate the experimentally measured data from a mechanical test, named the multi-relaxation (MR) test [26]. The value range for the fitting parameters is then used to discuss the role of glass fiber on the viscous stress response of the PC matrix in GF-PC.

## 2. Materials and Methods

### 2.1. Multi-Relaxation Test

The MR test used in this study consists of multiple stages of loading and then relaxation at different specimen displacements (based on the stroke of the test machine). All MR tests were conducted using a universal test machine, Quasar 100, at ambient temperature (about 23 °C). The tests were conducted using a common setup for tensile testing of materials, including a set of specimen holder, a load cell to measure the applied force and a desktop computer for test control and data recording. The specimens were gripped using a torque wrench to ensure consistency of the gripping force to avoid slippage during testing. At least two specimens were tested for each material to examine the repeatability of the test results.

**Figure 1:**
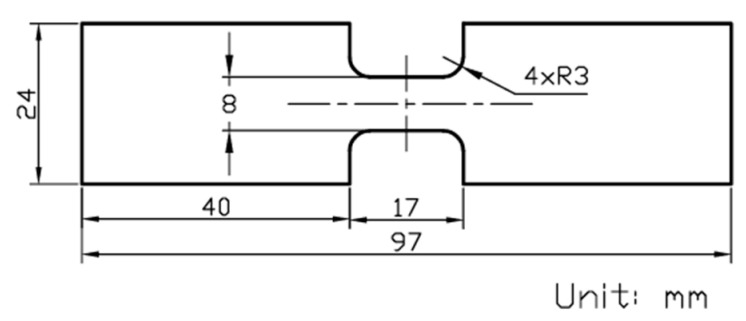
Dimensions of specimens used in the MR test.

Commercial plates of pure PC and GF-PC, of 9.3 and 11 mm thick, respectively, were provided by McMaster Carr, with the latter containing 20 wt.% of short glass fiber. Dog-bone specimens were water-jetted from the plates. Details of the specimen geometry and dimensions are shown in Figure 1.

**Figure 2:**
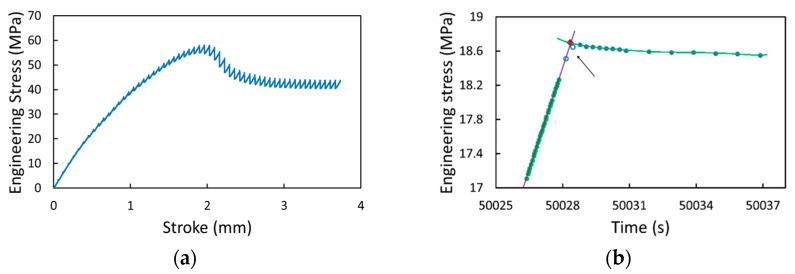
Examples of the MR test results: (**a**) engineering stress–stroke curve; (**b**) schematic depiction for the determination of the time for the onset of relaxation (in this case, at a stroke of 0.387 mm). These curves were from a pure PC specimen.

As an example of the data collected from the MR test, Figure 2a presents an engineering stress–stroke curve for a pure PC specimen. The stroke increment at each loading stage was 0.065 mm, introduced at the crosshead speed of 1 mm/min (i.e., 0.0166 mm/s), which was followed by a relaxation period of 10,000 s with the stroke unchanged. Details of the MR test methodology and data acquisition are presented in Ref. [26]. For this study, however, the MR tests were terminated when the specimens either fractured or showed a steady level of engineering stress with the increase in the stroke, i.e., with little change in the maximum stress over several loading stages.

Figure 2b shows the plot of engineering stress versus time for a pure PC specimen at the transition from a loading stage to the following relaxation stage at the stroke of 0.387 mm. The figure serves as an example of the procedure used to determine the point for the onset of relaxation. As shown in Figure 2b, the point of the onset of relaxation is at the intersection of the two fitting curves, one being a straight line that fits the last 30 points at the loading stage and the other a curve of the 6th-order polynomial function that fits the first part of the stress decay at the relaxation stage. A 6th-order polynomial function was chosen to fit the points at the beginning of relaxation because this is the lowest-order polynomial function that provides a good fitting at the beginning of relaxation for all strokes covered in this study. Polynomials of a lower order could show poor fitting at some strokes.

Note that in the determination of the point of the onset of relaxation, the first two points recorded at the relaxation stages, as indicated by an arrow in Figure 2b, were not included in the curve-fitting process. This is because the stresses at these two points were still increasing with the increase in time, contradictory to the common concept that at relaxation, stress should decrease monotonically with the increase in time. Such a stress increase was observed for all pure PC and GF-PC specimens used in this study, but to our knowledge, this phenomenon was never reported in the literature, such as the data shown in Refs. [7,27]. We believe that failing to detect the stress increase at the beginning of relaxation was because of the low sampling frequency used in those works, which was in the range from every 2 to 100 s, whereas the sampling frequency used in the current study at the beginning of relaxation was for every 0.33 s in order to capture the fast rate of stress change at the beginning of relaxation. The stress increase at the beginning of relaxation is believed to be an inertia effect, though a further study would be needed to fully understand the mechanism that is responsible for the stress increase.

### 2.2. Three-Branch Model

**Figure 3:**
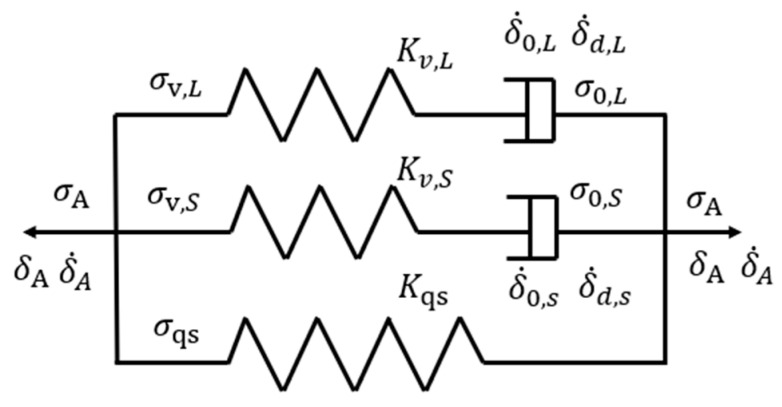
The three-branch model used in this study.

Stress decay as a function of time, at various strokes from the MR tests, was analyzed using a three-branch model shown in Figure 3. In this figure, the bottom branch contains only a spring and is used to represent the quasi-static component of the stress generated from the tests. The top two branches, each with a spring and a dashpot connected in series (i.e., the Maxwell branch), represent the viscous stress counterpart. A two-branch model, with one spring branch and one Maxwell branch connected in parallel, is a classical model for the simulation of the relaxation behavior of polymers [28]. However, as shown in our previous work, the two-branch model is insufficient for simulating the highly nonlinear stress drop of PE at the relaxation stage [29]. The three-branch models with two Maxwell branches, on the other hand, could accurately reproduce the stress response of polymers [25,30,31]. Therefore, the three-branch spring–dashpot model is selected for the data analysis.

As a model with three branches connected in parallel, the applied stress ($\sigma_{A}$) introduced in the MR test is represented by the sum of stresses applied to the three branches, i.e.,

(1) $$\sigma_{A}\left(t\right)=\sigma_{qs}+\sigma_{v,S}\left(t\right)+\sigma_{v,L}(t)$$

where $\sigma_{qs}$ is the quasi-static stress, $\sigma_{v,S}(t)$ the viscous stress in the short-term branch (the middle branch in Figure 3) and $\sigma_{v,L}(t)$ the viscous stress in the long-term branch (the upper branch in Figure 3). Subscripts ‘S’ and ‘L’, which will be applied hereafter, represent ‘short-term’ and ‘long-term’ branches, respectively.

Stress applied to the dashpots ($\sigma_{v}$) in the three-branch model is governed by the Eyring’s law [32]. In this study, deformation is represented by displacement $\left(\delta \right)$, and for the dashpot, the deformation rate (${\dot{\delta }}_{d}$) and the viscous stress follow the relationship below:

(2) $$\sigma_{v,S}/\sigma_{0,S}\;=\;{sinh}^{-1}\left({\dot{\delta }}_{d,S}/{\dot{\delta }}_{0,S}\right)$$

(3) $$\sigma_{v,L}/\sigma_{0,L}={sinh}^{-1}\left({\dot{\delta }}_{d,L}/{\dot{\delta }}_{0,L}\right)$$

where $\sigma_{0}$ is the reference stress and ${\dot{\delta }}_{0}$ the reference deformation rate.

Note that the summation of the displacement applied to the dashpot ($\delta_{d}$) and the displacement applied to the spring ($\sigma_{v}/K_{v}$) in the same branch (also referred to as the stroke to provide a meaning similar to the stroke introduced by the test machine) is equal to the applied stroke ($\delta_{A}$), which is assumed to be equivalent to the stroke of the test machine, i.e., by ignoring the displacement in the specimen section between the end of the gauge section and the grip of the test machine. Since $\delta_{A}$ is fixed during relaxation, ${\dot{\delta }}_{d}$ should have a magnitude equivalent to the stroke rate of the spring (${\dot{\sigma }}_{v}/K_{v}$) in the same branch, but in the opposite direction. That is,

(4) $${\dot{\delta }}_{0,S}\sinh\left(\sigma_{v,S}/\sigma_{0,S}\right)+{\dot{\sigma }}_{v,S}/K_{v,S}=0$$

(5) $${\dot{\delta }}_{0,L}\sinh\left(\sigma_{v,L}/\sigma_{0,L}\right)+{\dot{\sigma }}_{v,L}/K_{v,L}=0$$

The expression for the measured stress decay $\left(\Delta \sigma_{v}\right)$ during relaxation can be expressed as the sum of the stress decays from the short- and long-term branches, as shown in Equation (6). Details of the derivation are given in Ref. [28] and thus not repeated here.

(6) $$\begin{array}{c} \Delta \sigma_{v}=\sigma_{v,S}\left(0\right)-2\sigma_{0,S}{tanh}^{-1}\left[\tanh\left(\sigma_{v,S}\left(0\right)/2\sigma_{0,S}\right)\exp\left(-t/\tau_{v,S}\right)\right]\\ +\sigma_{v,L}\left(0\right)-2\sigma_{0,L}{tanh}^{-1}\left[\tanh\left(\sigma_{v,L}\left(0\right)/2\sigma_{0,L}\right)\exp\left(-t/\tau_{v,L}\right)\right] \end{array}$$

where $\tau_{v}$ is the characteristic relaxation time, defined as:

(7) $$\tau_{v,s}=\sigma_{0,S}/\left({\dot{\delta }}_{0,S}K_{v,S}\right)$$

(8) $$\tau_{v,L}=\sigma_{0,L}/\left({\dot{\delta }}_{0,L}K_{v,L}\right)$$

In this study, values for the fitting parameters $\sigma_{v,S}\left(0\right)$, $\sigma_{0,S}$, $\tau_{v,S}$, $\sigma_{v,L}\left(0\right)$, $\sigma_{0,L}$ and $\tau_{v,L}$ in Equation (6) were determined using an inverse approach [33] so that the curves of the stress decay generated from Equation (6) agree well with the experimental measurements.

**Figure 4:**
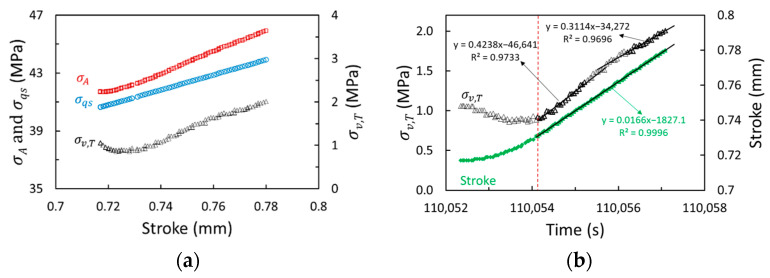
An example of the variation of stresses and stroke at a loading stage for GF-PC: (**a**) $\sigma_{A}$, $\sigma_{qs}$ and $\sigma_{v,T}$ as functions of stroke; (**b**) $\sigma_{v,T}$ and stroke as functions of time. The data are from a GF-PC specimen.

Values for spring moduli, $K_{v,S}$ and $K_{v,L}$, were determined through the analysis of the MR test data at the loading stages. Figure 4 presents an example of using data from a GF-PC specimen at the loading stage with the stroke increase from 0.72 to 0.78 mm to illustrate the analysis. Figure 4a shows $\sigma_{A}$, $\sigma_{qs}$ and total viscous stress $\left(\sigma_{v,T}\right)$ as functions of stroke at a loading stage, where

(9) $$\sigma_{v,T}\left(t\right)=\sigma_{A}\left(t\right)-\sigma_{qs}(t)$$

and Figure 4b shows the corresponding time functions of $\sigma_{v,T}$ and stroke. Two straight lines are used in Figure 4b to fit the different sections of the $\sigma_{v,T}$ curve after the rate of stroke increase reaches the programmed crosshead speed of 1 mm/min (0.0166 mm/s), i.e., after the time indicated by the vertical dashed line (red in the electronic version).

As shown in Figure 4b, the section of the $\sigma_{v,T}\left(t\right)$ curve after the rate of stroke increase reached 0.0166 mm/s could be approximated as two sections of straight lines, where the slope for the first straight line is 0.4238 MPa/s and the second is 0.3114 MPa/s. The critical time for the change in the slopes is around 110,056 s. The expressions of $K_{v,S}$ and $K_{v,L}$ are given below based on the distinct change in the slopes for the two straight lines, for which the detailed derivation is given in Ref. [34].

(10) $$K_{v,S}=[\sigma_{0,S}/(\tau_{v,S}{\dot{\delta }}_{d,S})]sinh(\sigma_{v,S}\left(0\right)/\sigma_{0,S})$$

(11) $$K_{v,L}=\left(1/{\dot{\delta }}_{A}\right)[{\dot{\sigma }}_{v,T,end}+(\sigma_{0,L}/\tau_{v,L})sinh(\sigma_{v,L}\left(0\right)/\sigma_{0,L})]$$

where ${\dot{\sigma }}_{v,T,end}$ is ${\dot{\sigma }}_{v,T}(t)$ at the end of the loading stage, at which ${\dot{\delta }}_{d,S}$ is equal to ${\dot{\delta }}_{A}$, i.e., 0.0166 mm/s [34].

### 2.3. Search Scopes for the Fitting Parameter Values

Three hundred sets of values for the six fitting parameters, $\sigma_{v,S}(0)$, $\sigma_{0,S}$, $\tau_{v,s}$, $\sigma_{v,L}(0)$, $\sigma_{0,L}$ and $\tau_{v,L}$, were determined using the genetic algorithm program in MATLAB R2023a. Criterion for ending the search for one set of values was when one of the following conditions was met, that is, either the number of generations reached 200 or the fitting errors were smaller than the predefined threshold. These sets of values were then sorted based on the difference between stresses generated from the model and those measured experimentally, from which 100 sets of values were selected, all of which yielded the maximum fitting errors of less than 0.08 MPa.

The genetic algorithm program used to search for the 300 sets of the fitting parameter values required an input of the limits for a value range, known as search scopes, for each of the six fitting parameters. In this study, the search scopes were chosen to cover a wide range of the fitting parameter values while keeping the calculation time within an acceptable limit of no more than 3 days for obtaining the 300 sets of the values at each relaxation stages for the entire MR test. For GF-PC, three trials were conducted to determine the appropriate search scopes, as presented in Table 1. The search scopes used in the first trial were based on the value ranges obtained by manual fitting. Two additional trials were conducted, which were to broaden the search scopes for the fitting parameters that had a significant number of values being very close to the search scope limits in the previous trial. Out of the three trials for GF-PC, the search scopes specified in the second trial were used to determine the 300 sets of the fitting parameter values for the analysis, as the third trial did not show much improvement in the distribution of the values within the search scope limits. For pure PC, the search scopes used in the first trial actually generated a reasonable distribution of the fitting parameter values and thus no additional trials were conducted.

Note that in view of the overlap of the search scopes for $\tau_{v,S}$ and $\tau_{v,L}$, a constraint was introduced in the genetic algorithm program to ensure that $\tau_{v,L}$ was always larger than $\tau_{v,S}$ in each set of the fitting parameter values.

## 3. Test Results and Analysis

### 3.1. Multi-Relaxation Test Results

**Figure 5:**
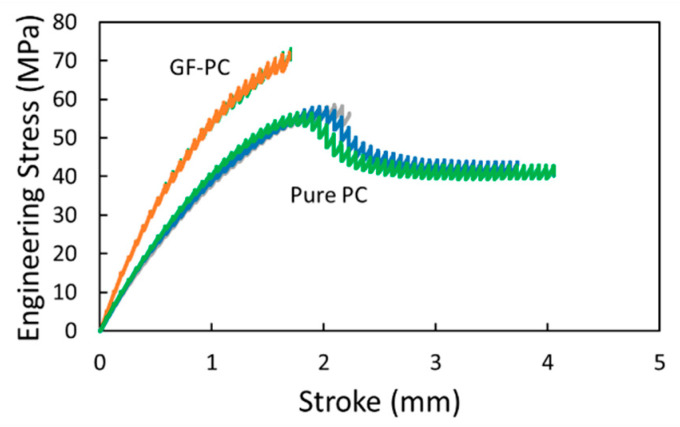
Engineering stress–stroke curves for pure PC and GF-PC specimens.

All engineering stress–stroke curves obtained from the MR tests are summarized in Figure 5, which shows good consistency among the curves for the same material, with the initial slope of the curves for GF-PC being larger than that for pure PC. The figure also indicates that GF-PC specimens broke before reaching a peak stress, in a brittle manner and at a stroke around 1.7 mm. For pure PC specimens, all curves contain a peak point at similar stress levels, but one specimen broke at the stroke about 2.2 mm while the others generated a plateaued stress level after the peak point and without showing any sign of fracture initiation at the end of the tests, at a stroke around 3.7 or 4.2 mm. Therefore, the early fracture of the pure PC specimen was believed to be caused by the presence of a defect in the gauge section. Overall, Figure 5 suggests that the pure PC specimens are much more ductile than the GF-PC specimens.

In view of the consistency of the test results, data analysis presented here is for one specimen of GF-PC and one of pure PC, the latter selected from the two specimens without the premature fracture. In addition, since the maximum stress at the loading stages and the stress decay at the relaxation stages for the pure PC specimens remained virtually unchanged after the strokes reached 2.88 mm, data analysis for the chosen pure PC specimen was conducted only up to the stroke of 2.88 mm.

### 3.2. Fitting Parameters in the Three-Branch Model

**Figure 6:**
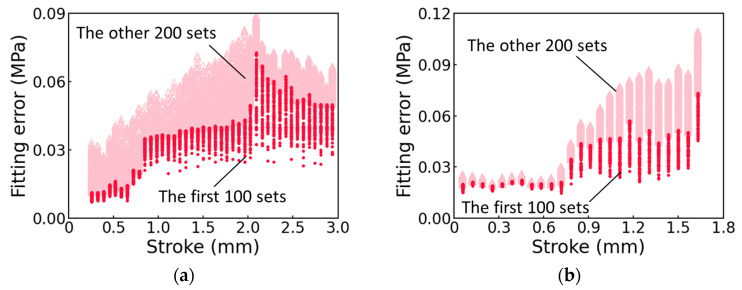
Comparison of the fitting errors for (**a**) pure PC; (**b**) GF-PC. The fitting errors for the first 100 sets of the fitting parameter values are marked using the solid circles (red in the electronic version).

Figure 6a,b for pure PC and GF-PC, respectively, summarize the fitting errors for the 300 sets of the fitting parameter values as a function of stroke. As mentioned earlier, among the 300 sets of fitting parameter values, the 100 sets that have the smallest fitting errors were selected for further analysis, for which the fitting errors are presented in Figure 6 using the solid circles and labeled as ‘the first 100 sets’. Note that the fitting errors are less than 0.08 MPa for the first 100 sets of the fitting parameter values, while those reported in the literature [19,20,21], were in the range from 0.15 to 1 MPa.

**Figure 7:**
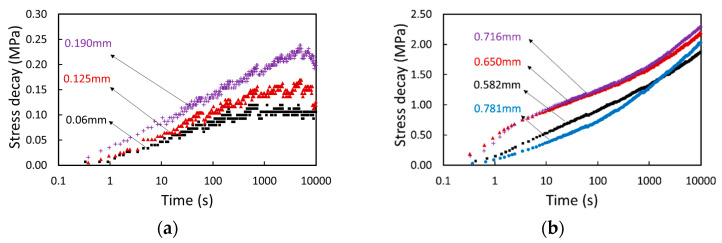
Summary of the stress decay curves that were not considered in the analysis: (**a**) the entire stress decay curves for the pure PC specimen; (**b**) the first 10 s of the stress decay curves at the strokes of 0.582, 0.65 and 0.716 mm for the GF-PC specimen. The stress decay curve at the stroke of 0.781 mm in (**b**) is to depict the typical curve profile that could be well fitted using the three-branch model.

It should also be noted that some experimental data from the MR tests have poor quality and are thus not used in the analysis. For the pure PC specimen, as shown in Figure 7a, the stress decay curves obtained from the first three relaxation stages did not show a sufficient resolution to provide a proper quantification of the fitting errors, some of which even showed a decrease in the stress decay before the end of relaxation. As a result, these curves were excluded from the analysis. Similar phenomena were also observed in the data from the other two pure PC specimens.

For the GF-PC specimen, as shown in Figure 7b for the strokes of 0.582, 0.65 and 0.716 mm, the stress decay curves within the first 10 s could not be properly fitted using the three-branch model. For comparison, Figure 7b also includes a stress decay curve at the stroke of 0.781 mm, which represents the typical curve profile that could be well fitted using the three-branch model. The main difference of the former three stress decay curves from the typical curve profile lies in the data for the first 10 s of relaxation. When this part of the stress decay curve could not be fitted using the three-branch model, the stress decay data for the first 10 s were ignored in the analysis. A similar phenomenon was also observed at some relaxation stages for the other GF-PC specimen. Although ignoring these parts of the stress decay curves did not affect the trend of dependence of the fitting parameter values on the stroke, a further study would be needed to examine how to fit the stress decay data in the first 10 s of relaxation at these strokes.

**Figure 8:**
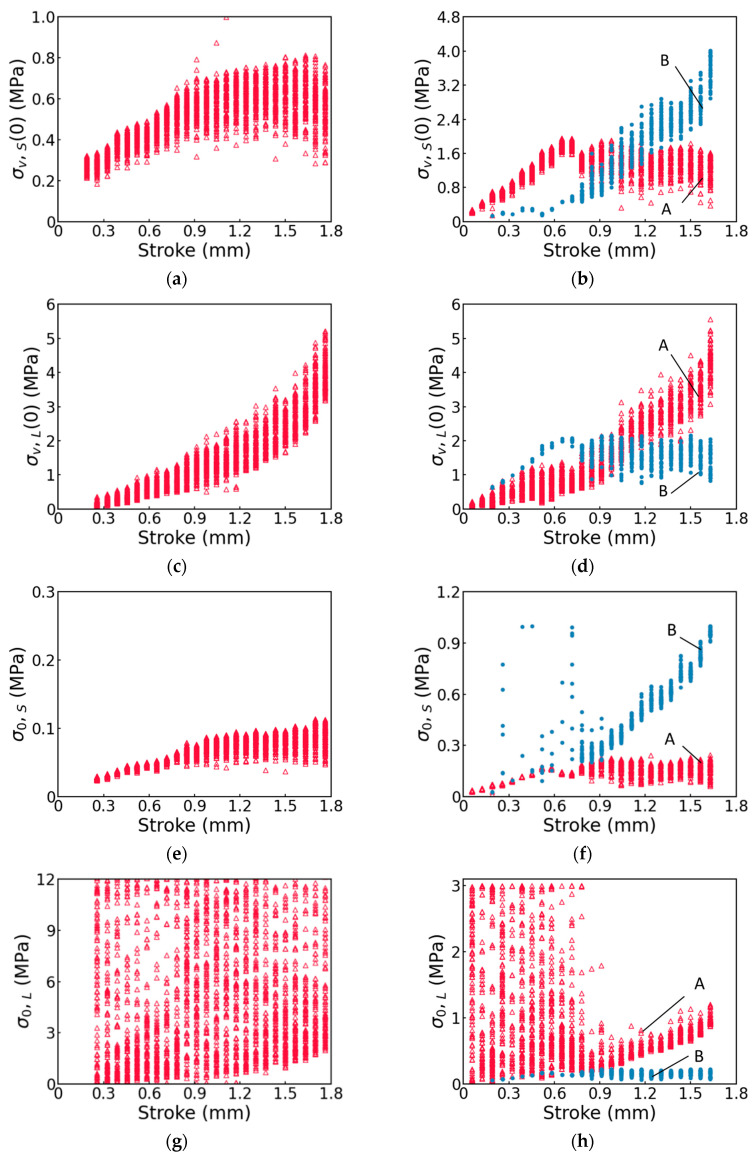

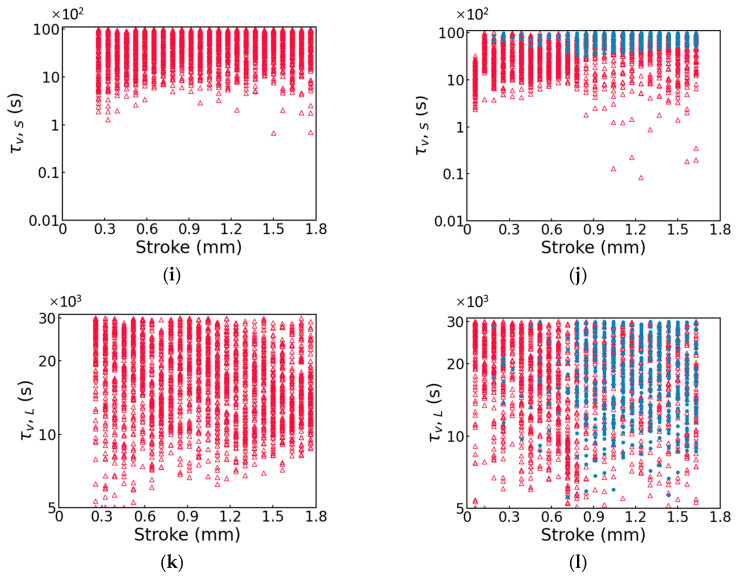
Summary of the values for the six fitting parameters in the three-branch model in order to fit the stress decay curves at relaxation: the left column (**a**,**c**,**e**,**g**,**i**,**k**) for the pure PC specimen and the right column (**b**,**d**,**f**,**h**,**j**,**l**) for the GF-PC specimen. For the GF-PC specimen, the open triangles (red in the electronic version) represent group A and the solid circles (blue in the electronic version) represent group B.

One hundred sets of values for $\sigma_{v,S}(0)$, $\sigma_{0,S}$, $\tau_{v,s}$, $\sigma_{v,L}(0)$, $\sigma_{0,L}$ and $\tau_{v,L}$, all of which could enable the three-branch model to mimic the stress decay at the relaxation stages of the MR test, are summarized in Figure 8, with the plots for the pure PC specimen on the left column and those for the GF-PC specimen on the right column. For the ease of comparison between the pure PC and GF-PC, plots of the fitting parameter values for the pure PC specimen are presented for the stroke only up to 1.8 mm, at which point the GF-PC specimen has already fractured.

Note that for the GF-PC specimen, the values for the fitting parameters $\sigma_{v,S}(0)$, $\sigma_{0,S}$, $\tau_{v,s}$, $\sigma_{v,L}(0)$, $\sigma_{0,L}$ and $\tau_{v,L}$ are divided into two groups, A and B, based on the corresponding $K_{v,S}$ values. Details for assigning the fitting parameter values to the two groups are given below.

**Figure 9:**
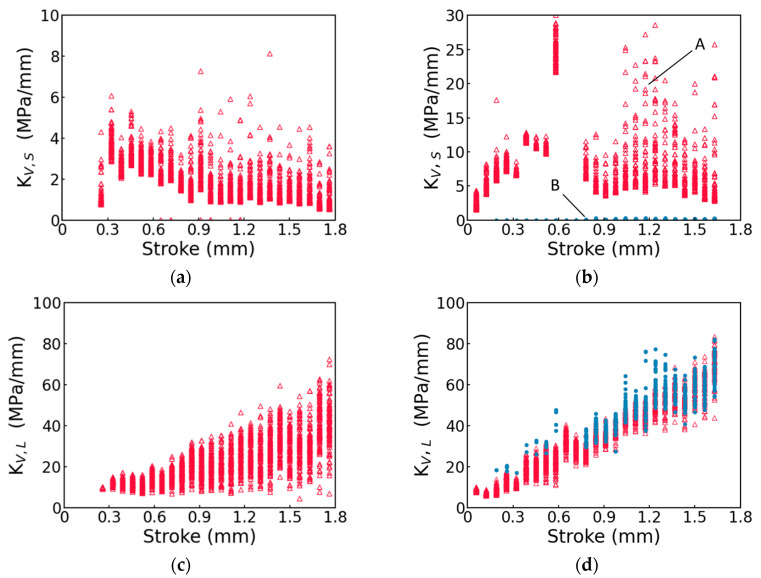
Summary of the spring moduli, $K_{v,S}$ and $K_{v,L}$, as functions of stroke: (**a**,**c**) pure PC; (**b**,**d**) GF-PC. For the GF-PC specimen, open triangles (red in the electronic version) represent group A and solid circles (blue in the electronic version) represent group B.

First of all, the spring moduli $K_{v,S}$ and $K_{v,L}$ for the two viscous branches, as summarized in Figure 9, were calculated using Equations (10) and (11) based on the six fitting parameters in Figure 8. For the pure PC specimen, as shown in Figure 9a, only 10 out of 2400 points have $K_{v,S}$ values close to zero. Since this is a very small fraction of the total number of data points, these near-zero $K_{v,S}$ values were ignored. After ignoring these 10 $K_{v,S}$ values, Figure 9a suggests that the $K_{v,S}$ values for the pure PC specimen form a single group at each stroke. On the other hand, for the GF-PC specimen, Figure 9b indicates that the $K_{v,S}$ values can be divided into two groups, A and B, with $K_{v,S}$ values in the former group ranging from 1 to 30 MPa and those in the latter group being close to zero. The corresponding fitting parameter values in Figure 8 that were used to determine the two groups of $K_{v,S}$ values were then also labeled as A and B.

The fitting parameter values in group B are believed to be just the mathematical solutions for the three-branch model to fit the stress decay observed in the GF-PC specimen, as these near-zero $K_{v,S}$ values across the entire stroke range suggest that this spring did not have any meaningful contribution to the stiffness of the short-term branch. That is, the spring could be removed from the short-term branch of the three-branch model. Since the total deformation of each branch remained constant at relaxation, without the presence of a spring, the deformation of the dashpot in the short-term branch could not change during relaxation, and thus the short-term branch could not contribute to the stress decay. That is, the fitting parameter values in group B of Figure 8 could not give any physical meaning for the behavior of the GF-PC specimen. Consequently, only the fitting parameter values in group A were used in this study to compare with the corresponding fitting parameter values for the pure PC specimen in order to examine the possible existence of any correlation of stress decay between the two materials.

**Figure A1:**
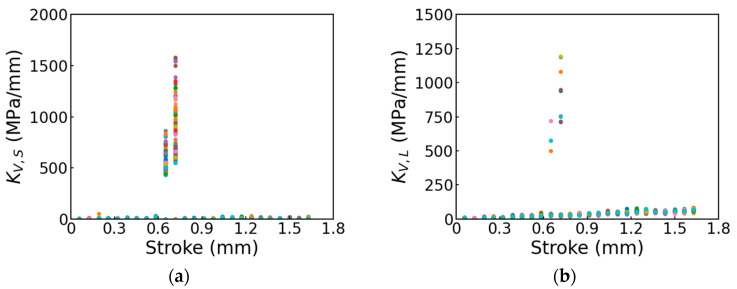
Spring modulus as functions of stroke in the three-branch model for the GF-PC specimen, including the extremely large values at the strokes of 0.65 and 0.716 mm: (**a**) spring modulus in the short-term branch ($K_{v,S})$; (**b**) spring modulus in the long-term branch ${(K}_{v,L})$.

Note that in addition to the $K_{v,s}$ values presented in Figure 9b for the GF-PC specimen, there were also some very large $K_{v,s}$ values, in the range of 200–2000 MPa/mm, determined based on the fitting parameter values at strokes of 0.65 and 0.716 mm in Figure 8, as shown in Figure A1 Appendix A. Since these values are far above the value range for the majority of the $K_{v,s}$ values, as shown in Figure 9b, these large values are deemed to be abnormal, though further study is needed to clarify the cause for the abnormality, which is being investigated while this manuscript is prepared.

As suggested in Figure 8a–h, the four fitting parameters, i.e., $\sigma_{v,S}(0)$, $\sigma_{v,L}\left(0\right)$, $\sigma_{0,S}$ and $\sigma_{0,L}$, for the GF-PC specimen in group A, show similar profiles to the corresponding four fitting parameters for the pure PC specimen, with the values for $\sigma_{v,S}(0)$ and $\sigma_{0,S}$ increasing initially with the increase in stroke and then reaching a plateau, and the values for $\sigma_{v,L}(0)$ and the lower bound of $\sigma_{0,L}$ increasing monotonically with the increase in stroke till the end of the MR test. It supports that the fitting parameter values in group A for the GF-PC specimen represent the viscous behavior of the matrix in GF-PC. On the other hand, as suggested earlier, the fitting parameter values for the GF-PC specimen in group B are just to provide the fitting function required to mimic the stress decay curves, not representing the viscous behavior of the matrix.

By comparing $\sigma_{v,S}(0)$ values for the pure PC specimen and group A of the GF-PC specimen, as shown in Figure 8a,b, respectively, the presence of glass fiber increased the short-term viscous stress for the PC matrix. The corresponding $\sigma_{v,L}(0)$ values, on the other hand, as shown in Figure 8c,d, were not affected by the presence of the glass fiber. However, the universality of this phenomenon needs further investigation using different material systems.

**Figure A2:**
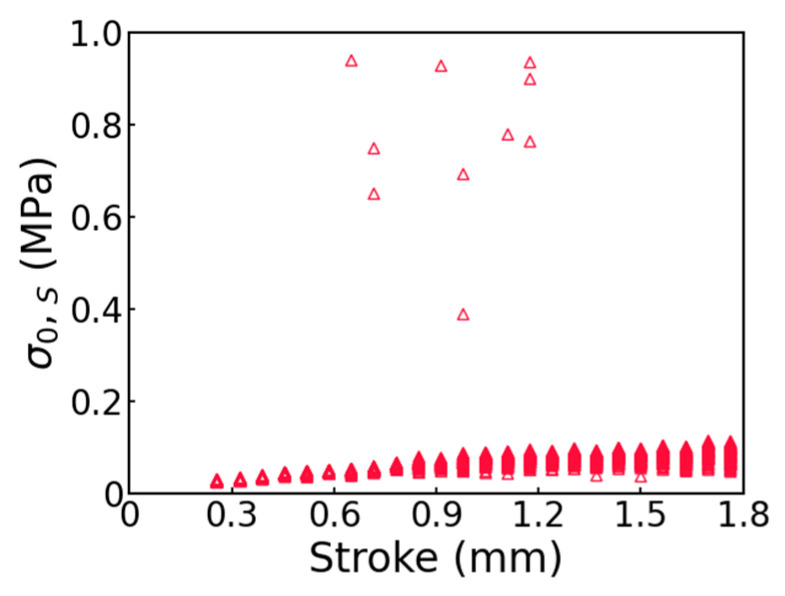
All 2400 points of $\sigma_{0,S}$ for the pure PC specimen, expressed as a function of stroke, including the 10 values that are much larger than the other data.

In addition to the above phenomena, data in Figure 8e and in group A of Figure 8f suggest that the presence of glass fiber does not have much influence on the variation of $\sigma_{0,S}$ with the increase in the stroke, suggesting that the rate of the stress decay in the short-term branch, which was mainly governed by the $\sigma_{0,S}$ value, was not much affected by the presence of glass fiber. Note that Figure A2 in Appendix A presents $\sigma_{0,S}$ values for pure PC, which includes the 10 data points that are ignored in Figure 8e.

Figure 8i–l shows that $\tau_{v,s}$ and $\tau_{v,L}$, for both pure PC and GF-PC specimens show relatively uniform scattering in the search scopes and did not provide any clear indication of a pattern for their dependence on the stroke increase.

As shown in Figure 9b for the GF-PC specimen, the $K_{v,s}$ values in group A are in the range of 1–30 MPa/mm, while the corresponding value range in Figure 9a, for the pure PC specimen, is much smaller, at 1–8 MPa/mm. However, these two figures show a similar profile of variation, suggesting that the presence of glass fiber enhances the resistance to the short-term viscous deformation of the GF-PC specimen. On the other hand, the increase in both the lower and the upper bounds of $K_{v,L}$ from Figure 9c to Figure 9d suggests that the presence of glass fiber enhanced the resistance to the long-term viscous deformation. However, the increase in the lower bound is much more significant than the increase in the upper bound.

### 3.3. Separation of the Viscous Stress from the Quasi-Static Stress

**Figure 10:**
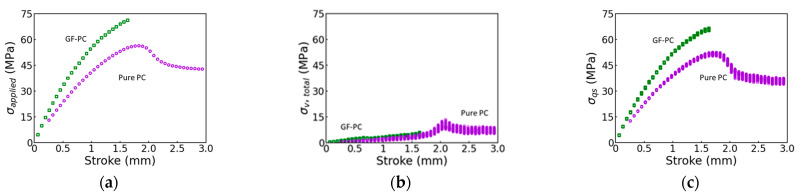
Summary of stresses determined from the MR tests as functions of stroke: (**a**) applied stress ($\sigma_{applied}$); (**b**) total viscous stress ($\sigma_{v,total}$); (**c**) quasi-static stress ($\sigma_{qs}$) of the GF-PC and pure PC specimens (green and purple, respectively, in the electronic version).

Figure 10 summarizes the values for the applied stress ($\sigma_{applied}$), the total viscous stress ($\sigma_{v,total}$, i.e., the sum of $\sigma_{v,L}\left(0\right)$ and $\sigma_{v,S}\left(0\right)$) and the quasi-static stress ($\sigma_{qs}$) for the pure PC and the GF-PC specimens as functions of the stroke. Figure 10a,c suggest that the GF-PC specimen shows a much higher applied stress and quasi-static stress than the pure PC specimen at the same stroke, while Figure 10b suggests that the total viscous stress of the GF-PC specimen is only slightly higher than that of the pure PC specimen, possibly because a relatively small fraction of the applied stress comes from the viscous component. In addition, as shown in Figure 8b,d, the influence of the glass fiber on the viscous stress of the GF-PC specimen is mainly seen in the increase in viscous stress in the short-term branch, as its influence on the long-term branch is much less significant.

## 4. Conclusions

The viscous behavior of GF-PC and pure PC was characterized using the MR test for the mechanical testing and a three-branch, spring–dashpot model for the analysis. One hundred sets of values for the fitting parameters were determined based on a genetic algorithm in MATLAB R2023a, all of which enabled the three-branch model to very closely regenerate the stress decay data at relaxation.

Using the above approach, the study has successfully separated the viscous stress response to deformation from the quasi-static counterpart. In addition, the study found that values of $\sigma_{v,S}(0)$ and $\sigma_{v,L}\left(0\right)$ showed a strong correlation between the pure PC and the GF-PC specimens, based on which the influence of the glass fiber on the viscous stress response of the PC matrix in GF-PC could be characterized.

Overall, the study concludes that the presence of short glass fiber enhances both the quasi-static and viscous stresses of the material. Furthermore, for the viscous stress, the presence of glass fiber is mainly to enhance the short-term viscous stress component. The influence of glass fiber on the long-term viscous stress is much smaller.

## Figures and Tables

**Table 1:** Search scopes for the fitting parameter values used in this study.

Parameters	Unit	GF-PC			PC
		First Trial	Second Trial	Third Trial	
$\sigma_{v,S}\left(0\right)$	MPa	[0, 2]	[0, 5]	[0, 5]	[0, 2]
$\sigma_{0,S}$	MPa	[0, 1]	[0, 1]	[0, 1]	[0, 1]
$\tau_{v,S}$	s	[0, 10,000]	[0, 10,000]	[0, 10,000]	[0, 10,000]
$\sigma_{v,L}\left(0\right)$	MPa	[0, 20]	[0, 20]	[0, 20]	[0, 20]
$\sigma_{0,L}$	MPa	[0, 3]	[0, 3]	[0, 12]	[0, 12]
$\tau_{v,L}$	s	[5000, 30,000]	[5000, 30,000]	[5000, 30,000]	[5000, 30,000]

## Data Availability

The data supporting the findings described in this manuscript are available from the corresponding authors upon request.
